# Sex and Gender-Related Differences in the Outcome of Total Hip Arthroplasty: A Current Concepts Review

**DOI:** 10.3390/medicina58121702

**Published:** 2022-11-22

**Authors:** Giuseppe Solarino, Davide Bizzoca, Anna Maria Moretti, Rocco D’Apolito, Biagio Moretti, Luigi Zagra

**Affiliations:** 1 Orthopaedics Unit, Department of Basic Medical Science, Neuroscience and Sensory Organs, School of Medicine, University of Bari “Aldo Moro”, AOU Consorziale Policlinico, 70124 Bari, Italy; 2 Ph.D. Course in Public Health, Clinical Medicine and Oncology, University of Bari “Aldo Moro”, Piazza Giulio Cesare 11, 70124 Bari, Italy; 3 Department of Pneumology, Santa Maria Hospital, Via De Ferrariis 18/D, 70124 Bari, Italy; 4 IRCCS Istituto Ortopedico Galeazzi, Hip Department, 20161 Milan, Italy; 5 Gruppo Italiano Salute e Genere (GISEG), 70126 Bari, Italy

**Keywords:** sex-related differences, gender-related differences, hip osteoarthritis, precision medicine, precision surgery, total hip arthroplasty (THA), total hip replacement (THR), robotic TH, robotic surgery

## Abstract

*Background and Objectives:* Sex and gender-related differences may influence the outcome of patients undergoing total hip arthroplasty (THA). The present paper aims to depict the importance of sex and gender-related issues in the perioperative management of patients undergoing THA to improve clinical outcomes and prevent postoperative complications. *Materials and Methods:* From January 2002 to August 2022, OVID-MEDLINE, EMBASE, SCOPU S, Web of Science, Google Scholar, and PubMed were searched to identify relevant studies for further analysis. The search strategy included the following terms: ((“gender-related differences” [MeSH Terms] OR “sex-related differences” [All Fields]) OR (“gender indicators” [MeSH Terms] OR “sex” [All Fields])) AND (“total hip arthroplasty” [MeSH Terms] OR (total hip replacement [All Fields])). *Results:* Twenty-eight papers were included in this current concepts review. Sex and gender-related differences were analyzed with regard to the following points: (1) surgical approach, robotic surgery, scar cosmesis, and implant choice; (2) postoperative clinical outcome and complications; (3) sexual activity after THA; and (4) psychological status and daily functional requirements. The data analysis showed that female patients need more specific attention in the preoperative, intraoperative, and postoperative phases to improve clinical and functional outcomes, reduce complications risk, and manage patient satisfaction. *Conclusions:* THA outcomes may be influenced by sex and gender-related factors which should be carefully assessed and addressed in patients undergoing surgery to improve the postoperative outcomes of patients’ satisfaction and reduce postoperative complications that can differ between the two sexes.

## 1. Introduction

Total hip arthroplasty (THA) has been defined as the operation of the 20th century, since its introduction to daily clinical practice has revolutionized the natural history of hip osteoarthritis, with very good long-term results reported [1].

THA surgery is currently one of the most common surgical procedures performed in orthopaedics and traumatology. The main indications for THA are primary and secondary hip osteoarthritis and intracapsular hip fractures [2,3].

According to the Registro Italiano ArtroProtesi (RIAP) 2020 report, 39,779 THAs were implanted in Italy in 2019 and 59.3% of these procedures were performed on female patients [3].

Despite improvements in surgical technique, perioperative patient management, and implant longevity, postoperative patient dissatisfaction is not uncommon in daily clinical practice [4]. Hence, it is reported that about 7% of patients are not satisfied with their postoperative outcomes [5].

The most common reasons for dissatisfaction after THA include painful THA, functional limitation, surgical complications, quality of care issues, slow recovery, and meeting patient expectations [4]. These data could be even more relevant in specific subsets of patients, considering that the female gender is an independent risk factor for readmission, reoperation, and wound infection after THA [6].

Based on these findings, it could be argued that sex and gender-specific analysis could be useful in preventing complications and improving clinical outcomes in patients undergoing THA.

Sex and gender-related factors influence health status throughout the lifespan, the pathogenesis of diseases, the response to pharmacologic and surgical treatment, and clinical outcome [7]. Sex indicates biological differences between women and men, mainly including gene expression, reproductive/sexual anatomy, hormone levels, and cyclic variation, that involve different anatomic and physiologic features [7,8]. Gender includes a complex and multidimensional concept involving several non-biological factors, including psychological aspects, sociocultural differences, education level, economic status, lifestyle, drug assumption, comorbidities, and religious beliefs [7,8].

This paper aims to highlight the importance of addressing sex and gender-related differences in the perioperative management of patients undergoing THA following precision medicine principles, to improve clinical outcomes and prevent postoperative complications and patient dissatisfaction.

## 2. Materials and Methods

The first step consisted of a scoping literature search performed on studies published from January 2002 to August 2022 by one reviewer, D.B., using the following databases: OVID-MEDLINE, EMBASE, SCOPUS, Web of Science, Google Scholar, and PubMed. We selected an initial pool of potentially relevant studies, aiming at investigating sex and gender-related issues in THA surgery.

The search strategy included the following terms: ((“gender-related differences” [MeSH Terms] OR “sex-related differences” [All Fields]) OR (“gender indicators” [MeSH Terms] OR “sex” [All Fields])) AND (“total hip arthroplasty” [MeSH Terms] OR (total hip replacement [All Fields])).

The second step consisted of revising the records to identify studies dealing with the impact of sex and gender-related differences in the outcome of THA. Inclusion criteria were clinical trials and systematic reviews focusing on sex and gender-related differences in patients undergoing THA, and papers written in English. The database search provided a total of 77 studies for potential inclusion in the review. After adjusting for duplicates, 39 studies remained. Of these, 13 studies were discarded after reading titles and reviewing abstracts. Two additional abstracts were identified by checking the references of the relevant papers. A total of 28 articles were finally included in the present review [4,8,9,10,11,12,13,14,15,16,17,18,19,20,21,22,23,24,25,26,27,28,29,30,31,32,33,34].

## 3. Results

### 3.1. Surgical Approach, Robotic Surgery, Scar Cosmesis, and Implant Choice

#### 3.1.1. Surgical Approach

In recent years, the increasing diffusion of the tissue-sparing surgery concept has promoted the development of several minimally invasive approaches for THA surgery, including the minimally invasive anterolateral approach (MAA), direct anterior approach (DAA), mini-posterior and supercapsular percutaneously assisted approach (SuperPath^®^, Microport- Shangai, China) [9,10,35]. Minimally invasive approaches aim at reducing soft tissue and muscular damage, enhancing postoperative recovery, and reducing surgical scar length and impact on patients’ aesthetic perception [9,10]. However, despite the growing interest in this topic, we found only one study [11] specifically focused on gender-related differences in a minimally invasive approach. Von Roth et al., in a prospective study recruiting 48 patients undergoing THA, investigated the impact of BMI and gender on the accuracy of THA positioning through a minimally invasive approach [11]. These authors observed comparable functional and radiological results in both groups, thus highlighting that BMI and gender do not influence THA positioning accuracy [11] (Table 1). It should be noted, however, that skeletal muscle has structural and hormone-related differential features in female patients compared to male patients [12], therefore each surgical approach may cause a different recovery and postoperative outcome in both sexes; this relationship should be further investigated in future clinical trials.

#### 3.1.2. Robotic Surgery

Over the last decade, robotic-assisted THA has gained increasing popularity in daily clinical practice to reduce surgical error and improve the accuracy of implant positioning, thus restoring better hip biomechanics compared to the conventional manual THA [13,36]. Based on the abovementioned points, a robotic THA should hopefully lead to further improvements in functional outcomes and implant survivorship in the near future [13]. Nonetheless, McDermott et al. [14], in a survey-based study recruiting 25 patients (12 males and 13 females) from different social/ethnic backgrounds, investigated the male and female perceptions of robot-assisted surgery, mainly focusing on factors that might facilitate or inhibit its acceptance. In this study, most female participants expressed concerns about robotic surgery safety, whereas male participants reported being unfazed by robotic-assisted procedures [14] (Table 1). The data also showed clear differences in how males and females perceived and conceptualized robot-assisted surgery, thus highlighting the need to develop sex-specific preoperative strategies to describe the surgical procedure, to better comfort the patient preoperatively.

#### 3.1.3. Scar Cosmesis

The surgical scar and its subjective perception are other key points in determining a patient’s satisfaction with the surgical procedure. Lari et al. [15], in an online survey recruiting 303 surgeons, investigated the importance of scar cosmesis across different specialties. Based on the results, scar cosmesis was rated lowest among orthopaedic surgeons, compared with plastic surgeons, obstetricians, and gynaecologists. Nonetheless, when considering patient demographics, surgeons mostly reported preferring scar cosmesis in young and female patients [15] (Table 1). These data support the increasing diffusion of minimally invasive approaches in THA surgery, aiming to improve postoperative patient satisfaction, the length of skin incision, and suture and wound care accuracy.

#### 3.1.4. Implant Choice

Hip resurfacing arthroplasty (HRA) with metal-on-metal (MoM) bearing surfaces gained popularity over the last two decades, but gender-related differences in implant survival and postoperative complications have been widely reported [16]. Haughom et al., in a systematic review analyzing ten papers, reported a higher complication rate in women undergoing MoM-HRA compared to men [16] (Table 1). Complications included adverse local tissue reaction, implant dislocation, aseptic loosening, and revision surgery [16]. It has been hypothesized that femoral head size could be a prime factor in women’s higher rate of complications, but this relationship has not yet been probed [17]. Suspected higher immunogenic response to metal ions in females could be a possible explanation of why, since even in the case of small metal-on-metal heads, women show a greater number of adverse reactions to metal debris (ARMD) [37].

### 3.2. Postoperative Clinical Outcome and Complications

Postoperative complications and functional outcomes play a central role in determining the patient’s quality of life after THA surgery.

Biological differences that influence native hip and THA biomechanics will surely influence postoperative functional outcomes. Peng et al. [18], in an observational study recruiting 33 patients (females: 69.7%), analyzed the effect of gender on gait asymmetry after unilateral THA (Table 2). These authors observed that females undergoing unilateral THA exhibited a significantly increased adduction of the THA compared to the native hip. This finding could be partially explained by considering a superior position of the femoral stem centre of rotation in females [18]. The understanding of such gender-specific differences in THA kinematic patterns may promote targeted preoperative planning and postoperative rehabilitation, thus improving postoperative outcomes [18].

It is also remarkable to note that Kostamo et al. [19], in a retrospective study analyzing 3461 consecutive patients receiving 4114 primary THAs (females: 55.6%) between 1980 and 2004, concluded that there is no apparent need for a gender-designed THA system (Table 2). These authors reported no significantly different survivorship or revision rate between the two sexes but they depicted that males used larger stems with greater stem lengths, neck offset, and neck lengths [19]. According to the author’s ‘opinion’, current implant systems are sufficiently versatile to address the different sizes and offset needs of male and female patients, hence gender-designed THA implants may be unnecessary in daily clinical practice [19]. However, preoperative planning and intraoperative component positioning must respect biological differences to reach a better postoperative clinical and functional outcome [19].

Sex-related factors may explain not only biomechanical differences but also functional outcome differences in patients undergoing THA. Holtzman et al. [20], in a prospective observational study recruiting 1120 patients older than 65 who underwent THA (female: 61.4%), examined differences in functional status and pain at the time of THA and 1-year follow-up (Table 2). At the time of THA, after adjusting for comorbidities and age, males could walk greater distances and be less likely to suffer from severe pain during walking (*p* < 0.01), to need assistance with walking (*p* < 0.01), housework (*p* < 0.01) and drugstore shopping (*p* < 0.001), compared with females [20]. At one year of follow-up, sex-related differences were still observed in the recruited patients: females could still only walk shorter distances and reported a high rate of required assistance for walking (*p* < 0.01), housework (*p* < 0.01) and drugstore shopping (*p* < 0.01) [20]. A study from the Dutch joint registry (6030 patients) that identified three subgroups of patients with different functional recovery trajectories after THA (fast starters (87.7%), slow starters (4.6%), and late dippers (7.7%)), showed that female patients tended to be slow starters [38] (Table 2).

Nonetheless, the functional outcome depends not only on biological factors but also on gender-related factors. Patel et al. [6], in a retrospective review of the American College of Surgeons National Surgical Quality Improvement Program (ACS-NSQIP), assessed gender-related differences—i.e., demographic data, comorbidity profiles, operative time, hospital length of stay (LOS), and 30-day outcomes—in 418,885 patients (females: 59.1%) who underwent elective THAs and TKAs between 2011 and 2017 (Table 2). In this study, compared with males, females showed significant differences (*p* < 0.01) in terms of higher body mass index (BMI), older age, and the need for preoperative functional assistance [6]. In contrast, when considering comorbidities, male patients showed higher rates of hypertension, diabetes, kidney disease, and anaemia, but a lower rate of chronic steroid assumption (*p* < 0.001), compared with female patients [6]. These authors finally depicted the female gender as an independent risk factor for wound infection, readmission, and reoperation following THA (*p* < 001) [6]. Females were also revealed 64–82% to be more likely to require a LOS > 2 days than males in this study [6]. These findings of course need to be carefully interpreted, as controversial results have been published in the past showing that the male sex has been associated with increased mortality, better functional outcome, no difference in pain, and increased risk of revision [39].

In a literature review, Sidler-Maier and Waddel [40] showed that the female gender together with advanced age and cementless implants were associated with a higher risk of periprosthetic fractures. More recently, a Norwegian Arthroplasty Register study based on 66,995 THAs from 2005 to 2017 also found that the highest risk of revision was in elderly women (≥55 years of age) receiving cementless stems, mainly due to periprosthetic fracture but also to dislocation [41]. Similar findings were shown by Lamb et al. [42] (Table 2) in a cohort of 793,823 primary THA between 2004 and 2016 in the National Joint Registry (NJA) database: female gender significantly increases the risk of intraoperative fracture. Intraoperative and early periprosthetic fracture is a severe complication that even if treated early, significantly increases the risk of revision within 10 years after surgery and the 90-day mortality compared to uncomplicated THA [43]. When choosing the type of implant and type of fixation, this must be taken into account in this selected population.

**Table 2:** Studies dealing with clinical outcomes and complications with regard to sex and gender.

Study	Design	Sample Size	Aim	Main Findings
Peng et al. [18]	Observational study	33 patients (female: 69.7%)	To investigate the effect of gender on gait asymmetry after unilateral THA.	Females undergoing unilateral THA exhibited significantly increased adduction of the THA compared to the native hip.
Kostamo et al. [19]	Retrospective study	3461 patients (females: 55.6%)	To investigate the survivorship and clinical outcomes of a primary THA cohort specifically assessing differences between genders in clinical outcomes, implant survivorship and revisions.	Survivorship and revision rates were not significantly different. Men used larger stems with greater stem lengths, neck offset, and neck lengths. Current implant systems were sufficiently versatile to address the different sizes and offset needs of male and female patients.
Holtzman et al. [20]	Prospective observational study	1120 patients (female: 61.4%)	To assess differences in functional status and pain in men and women at the time of THA and 12-month follow-up.	At the time of THA, males could walk greater distances and be less likely to suffer from severe pain during walking (*p* < 0.01) and need assistance with walking (*p* < 0.01), housework (*p* < 0.01) and drugstore shopping (*p* < 0.001), compared with females. At one year of follow-up, sex-related differences were still observed in the recruited patients.
Patel et al. [6]	Retrospective study	418,885 patients (females: 59.1%)	To assess gender-related differences in patients who underwent elective THAs and TKAs.	The female gender revealed an independent risk factor for wound infection, readmission, and reoperation, following THA (*p* < 0.001). Females revealed also 64–82% more likely to require a LOS >2 days than males.
Lamb et al. [42]	Retrospective study	793,823 (female: 61.18%)	To estimate risk factors for intraoperative periprosthetic femoral fractures during primary total hip arthroplasty.	Female gender significantly increases the risk of intraoperative fracture.

### 3.3. Sexual Activity after THA

Besides walking, hip osteoarthritis (HOA) may significantly affect several physical activities of everyday life, including sexual activity in both genders [21] (Table 3). Furthermore, nowadays, THA is also performed in young patients for whom satisfying sexual activity is important for their well-being and could constitute a major postoperative outcome [4,22,23]. Based on these findings, surgeons should be aware of this surgical goal and inform patients preoperatively about postoperative activity improvements and limitations [24].

Issa et al. [22] assessed patient and surgeon perspectives about the effects of THA on sexual activity in a systematic review including nine patient-based studies recruiting 1694 patients (mean age: 57 years; range: 17–98 years) and two studies evaluating the perspective of 337 surgeons (Table 3). These authors reported that 76% of patients considered HOA as the leading cause of their preoperative sexual problems [22]. After THA, 44% of patients reported improvements in sexual satisfaction, whereas an increased intercourse frequency was reported in only 27% of patients [22]. The mean time interval between surgery and return to sexual activity was four months post-THA [22]. Similar data were also reported by Neonakis et al. [25] (Table 3).

Rougereau et al. [21], in a single-centre prospective cohort study recruiting 101 consecutive patients who received a 6-month follow-up after THA, investigated the influence of age and sex in the timing and modalities of sexual activity resumption after THA. Only 49 patients out of 101 (48.5%) revealed being sexually active before surgery, therefore data analysis was performed on these patients only [21] (Table 3). Male patients resumed preoperative sexual activity more often compared to females, in terms of frequency (*p* = 0.02) and quality of sexual positions (*p* = 0.003). Moreover, while females resumed sexual activity on average 6 weeks post-THA, males regained sex activity during the first 3 weeks after surgery [21]. In both genders, the main difficulty reported during sexual activity was the fear of THA dislocation, which was related, in part, to insufficient preoperative information [21].

Regarding surgeons’ awareness of the importance of patients’ postoperative resumption of sexual activity after THA, Issa et al. [22] reported that 88% of surgeons rarely or never discuss sexual activity with their patients, and 61% believed that patients can resume sexual activity 1 month after THA (Table 3). These data highlight orthopaedics pay little attention to the patient’s post-THA sex life.

Neonakis et al. [25] also reported that patient education concerning postoperative expectations and sexual activity resumption is severely lacking in daily clinical practice (Table 3). Most surgeons offer little or no information on post-THA sexual activity.

These findings become even more important when considering the data reported by Charbonnier et al. [26] who investigated the post-THA relative risk of impingement and joint instability during sexual activities (Table 3). For this purpose, these authors performed a motion capture study with two volunteers, that executed 12 common sexual positions [26]. After data analysis, four sexual positions requiring high hip flexion (>95°) caused prosthetic impingements (and subsequent risk of posterior instability) in females [26]. Nonetheless, when considering male positions, bony impingements (associated with anterior instability) occurred only during one sexual position requiring a high degree of external rotation (>40°) combined with extension and adduction [26].

Future studies, specifically designed on post-THA sexual activities are required to evaluate the effects of THA surgery, implant choice, and surgical approaches on postoperative sexual restrictions [25]. Furthermore, orthopaedic surgeons must dedicate more time to explaining the impact of THA on postoperative sexual activity, avoiding patients’ false expectations, and, in the meantime, educating the patients.

### 3.4. Psychological Status and Daily Functional Requirements

Postoperative psychological status and patient satisfaction are largely influenced by the patient’s history, functional requirements, age, civil status, working status, and domestic housework.

De Caro et al. [28] recently highlighted the need for accurate preoperative patient information, to avoid false post-THA functional expectations. Therefore, when such expectations are not met, patients may be dissatisfied with the outcome of a technically successful procedure [28] (Table 4). These authors also depicted that psychological factors and mental status can affect the outcome and satisfaction of patients undergoing THA [28]. Strategies focused on rapidly identifying and treating these preoperative conditions must be considered to improve THA outcomes [26].

Different papers confirm patient’s recovery post-THA is influenced by several psychological aspects, such as depression, anxiety, resilience, and personality traits [27,28,29,30,31,32], and therefore special attention should be addressed to these risk factors to prevent negative postoperative outcomes.

Postoperative functional requirements mainly depend on a patient’s age and domestic load/ workload.

Peeters et al. [33], in a systematic review based on 49 papers, investigated the health status (HS) and health-related quality of life (HRQOL) of elderly patients undergoing surgery for hip fractures (Table 4). These authors reported that although HS and HRQOL improved in the first 6 months, most of them did not return to the pre-fracture HS level [33]. Female gender, together with other factors including the patient’s mental state, comorbidities, nutritional status, postoperative pain, length of hospital stay, and complications were associated with worse HS or HRQOL.

Truszczyńska et al. [34], in an observational study analyzing 54 patients (younger than 65 and still in employment; male: 57.3%) who underwent THA for HOA, reported that only 59.3% of patients returned to work post-THA. The patients who returned to work perceived their health status as better than the patients who did not resume work [34] (Table 4). The main reasons for not going back to work were not medical, since pain elimination and hip range of movement increasingly enabled patients to return to work [34]. Moreover, among patients that resumed work after surgery, 40.7% of them did not return to preoperative employment [34]. Considering the differences in employment, psychological status, and attitude during the pre- and postoperative phases, different gender satisfaction can be expected.

## 4. Conclusions

Sex and gender-related factors should be carefully assessed and addressed in patients undergoing THA to improve postoperative outcomes and patient satisfaction rates and to reduce postoperative complication rates. The preoperative assessment of patients undergoing THA should include a psychological evaluation, the patient-specific risk factors evaluation and correction, and the patient’s subjective perception of body image. Furthermore, patients’ postoperative functional requirements should also be evaluated before surgery and patients must be informed about the potential physical limitations in their daily routine. Special attention should be also given to potential sexual activities and limitations after THA, thus avoiding patients’ false expectations, and in the meantime, educating the patients, thus preventing sexual-related complications.

The principles of the gender-specific approach should be part of medical education so that future generations of surgeons can better apply them in daily clinical practice and planning, thus hopefully improving postoperative outcomes and patients’ satisfaction.

Future studies are still needed to better understand the relationship between sex and gender-related factors and postoperative outcome and satisfaction in patients undergoing THA. Orthopaedic clinical research should pay more attention to gender-specific indicators to develop gender-based risk-stratification models.

## Figures and Tables

**Table 1:** Studies investigating surgical approach, robotic surgery, scar cosmesis, and implant choice in relation to sex and gender.

Study	Design	Sample Size	Aim	Main Findings
Von Roth et al. [11]	Prospective Study	48 patients (male: 21; female: 27)	assess the influence of BMI and gender on surgical accuracy during minimally invasive surgery (MIS) for THA	BMI and gender do not appear to influence outcomes using MIS for THA
McDermott et al. [14]	Survey-based study	25 patients (12 males and 13 females)	to investigate male and female perceptions of robot-assisted surgery	most of the females expressed concerns related to robotic surgery safety, whereas male participants reported being unfazed by robotic-assisted procedures
Lari et al. [15]	Survey-based study	303 surgeons belonging to different specialties	to investigate the importance of scar cosmesis across different specialties	scar cosmesis was rated lowest among orthopaedic surgeons
Haughom et al. [16]	Systematic review	10 papers including 9296 patients (male: 6389; female: 2907)	To assess the complication rates between men and women undergoing Metal-on-Metal Hip Resurfacing Arthroplasty (MoM-HRA)	a higher complication rate in women undergoing MoM-HRA compared with men

**Table 3:** Studies investigating sexual activity after THA and gender differences.

Study	Design	Sample Size	Aim	Main Findings
Rougereau et al. [21]	Prospective study	101 patients (74.26%)	Investigated the influence of age and sex in the timing and modalities of sexual activity resumption after THA.	Male patients resumed preoperative sexual activity more often, compared to females. Females resumed sexual activity on average 6 weeks post-THA, and males regained sex activity during the first 3 weeks after surgery. In both genders, the main difficulty reported during sexual activity was the fear of THA dislocation, which was related in part to insufficient preoperative information.
Issa et al. [22]	Systematic review	9 patient-based studies recruit-ing 1694 patients (mean age: 57 years) and two studies evaluating the perspective of 337 surgeons	To assess patient and surgeon perspectives about the effect of THA on sexual activity.	76% of patients considered HOA as the leading cause of their preoperative sexual problems; after THA, 44% of patients reported improvements in sexual satisfaction. The mean time interval between surgery and return to sexual activity was 4 months post-THA.
Neonakis et al. [25]	Systematic review	16 papers recruiting 2391 patients	To investigate the success of this surgical procedure to ameliorate the sexual activity of patients.	Sex life improved after THA. Patient education concerning postoperative expectations and sexual activity resumption is severely lacking in daily clinical practice.
Charbonnier et al. [26]	Observational study	Motion capture study with 2 volunteers, that executed 12 common sexual positions).	To investigate the post-THA relative risk of impingement and joint instability during sexual activities.	In females, four sexual positions requiring high hip flexion (>95°) caused prosthetic impingements.

**Table 4:** Studies showing the role of psychological status and daily functional requirements.

Study	Design	Sample Size	Aim	Main Findings
De Caro et al. [28]	Prospective study	289 patients (female: 59%)	To assess psychological factors that could improve the postoperative outcome in patients undergoing THA or TKA.	Psychological factors and mental status in primary total hip and knee replacement can affect the outcome and patient satisfaction. Strategies focused on the identification and facing of these conditions must be considered to improve the outcome of total replacement.
Peters et al. [33]	Systematic review	49 papers recruiting	To investigate the health status (HS) and health-related quality of life (HRQOL) of elderly patients undergoing surgery for hip fractures.	Female gender, together with other factors, was associated with worse HS or HRQOL.
Truszczyńska et al. [34]	Observational study	54 patients (female: 42.7%)	To analyze factors influencing return to work after THA.	The patients who returned to work perceived their health status as better than the patients who did not resume work.

## Data Availability

Not applicable.
